# Comparison of three surgical approaches for cervicothoracic spinal tuberculosis: a retrospective case–control study

**DOI:** 10.1186/s13018-015-0238-0

**Published:** 2015-07-02

**Authors:** Hao Zeng, Xiongjie Shen, Chengke Luo, Zhengquan Xu, Yupeng Zhang, Zheng Liu, Xiyang Wang

**Affiliations:** Department of Spine Surgery, The Xiangya Hospital of Central South University, 87# Xiangya Road, Changsha, Hunan 410008 People’s Republic of China; Department of Spine Surgery, Hunan Provincial People’s Hospital, Changsha, Hunan 410005 People’s Republic of China

**Keywords:** Tuberculosis, Cervicothoracic, Surgery, Three approaches, Complication, Outcome

## Abstract

**Study design:**

This is a retrospective case–control study.

**Objectives:**

The surgical approaches to cervicothoracic spinal tuberculosis (CTSTB) were controversial. The aim of this research is to retrospectively compare the efficacy and feasibility of anterior-only (AO) approach, combined anterior and posterior (AP) surgeries, and posterior-only (PO) approach for the treatment of CTSTB.

**Methods:**

AO approach was undertaken in 20 patients (group A), AP fusion was carried out in 18 patients (group B), and PO surgery was performed in 21 patients (group C). Surgery duration, intraoperative blood loss, length of hospitalization, neurological status, kyphosis angle correction, loss of correction, and complications of the three groups were compared.

**Results:**

Three surgical approaches all improved the kyphosis deformity and neurological function significantly (*P* < 0.05). The mean loss of correction in group A in the final follow-up was higher than in groups B and C (*P* < 0.05), and the difference between groups B and C was not significant (*P* > 0.05). The mean operation time, blood loss, and hospitalization days in group B were greater than in groups A and C. Complications were most prevalent in group A, more in group B, and the least in group C.

**Conclusion:**

The AO approach surgery should be limitedly used for severe CTSTB. The AP approach had got satisfactory clinical and radiographic outcomes, but with larger trauma and more complications, which should be reservedly performed for mild CTSTB. Compared to traditional surgery, PO surgery can significantly improve clinical results and obviously relieve postoperative complications.

## Introduction

The past decades have witnessed the resurgence and even increasing of tubercular disease attributed to the acquired immunodeficiency syndrome pandemic, surge of immigration, and impoverished living and sanitary conditions. Spinal tuberculosis (STB), which is a common extra-pulmonary, is the most frequent and serious form of skeletal tuberculosis. However, cervicothoracic spinal tuberculosis (CTSTB) is uncommon, and the treatment of CTSTB is described as atypical case reports published as rarities in the mainstream academic journals [1].

The potent anti-tuberculosis therapy (ATT) and external immobilization still play an irreplaceable role in treatment of CTSTB in most cases. But when CTSTB is characterized by bone destruction, severe kyphosis deformity, large abscess (including cold abscess), and spinal cord compression, usually beyond the routine ATT function, surgical intervention will be imperative. To our knowledge, surgical treatment of CTSTB has rarely been reported [2–5].

However, surgical approaches to CTSTB were controversial, attributed to its particular and even more complex anatomic structure. The elimination of tuberculosis, removal of nerve compression, the correction of kyphosis, and reconstruction and maintenance of spine stability have become the main purpose of surgical treatment of CTSTB. The advantages of stand-alone anterior surgery for CTSTB include improved visualization, radical debridement, directly addressing the clinically relevant pathology, more extensive decompression, and reconstruction of the anterior column, theoretically leading to improving rates of bone fusion and getting perfect clinical outcome [6, 7]. But persistent maintenance of spine stability will be out of its range [7–9]. Combined anterior and posterior approaches have perfect clinical results except some inconvenient complications [10]. Increasingly popular posterior-only surgery labeled with good clinical efficacy and fewer complications is getting more and more attention [2, 11]. But some surgeons persisted that its merits would be eclipsed by its incomplete debridement and destruction of original healthy posterior column structure [12]. However, there is no report to compare the efficacy and feasibility of the above three surgical approaches in treatment of CTSTB.

In our study, we presented the clinical details of the three surgical approaches in treatment of 59 patients with CTSTB and retrospectively compared the clinical outcomes of the three surgeries, which may be conductive to selecting a proper management of CTSTB.

## Materials

**Fig. 1:**
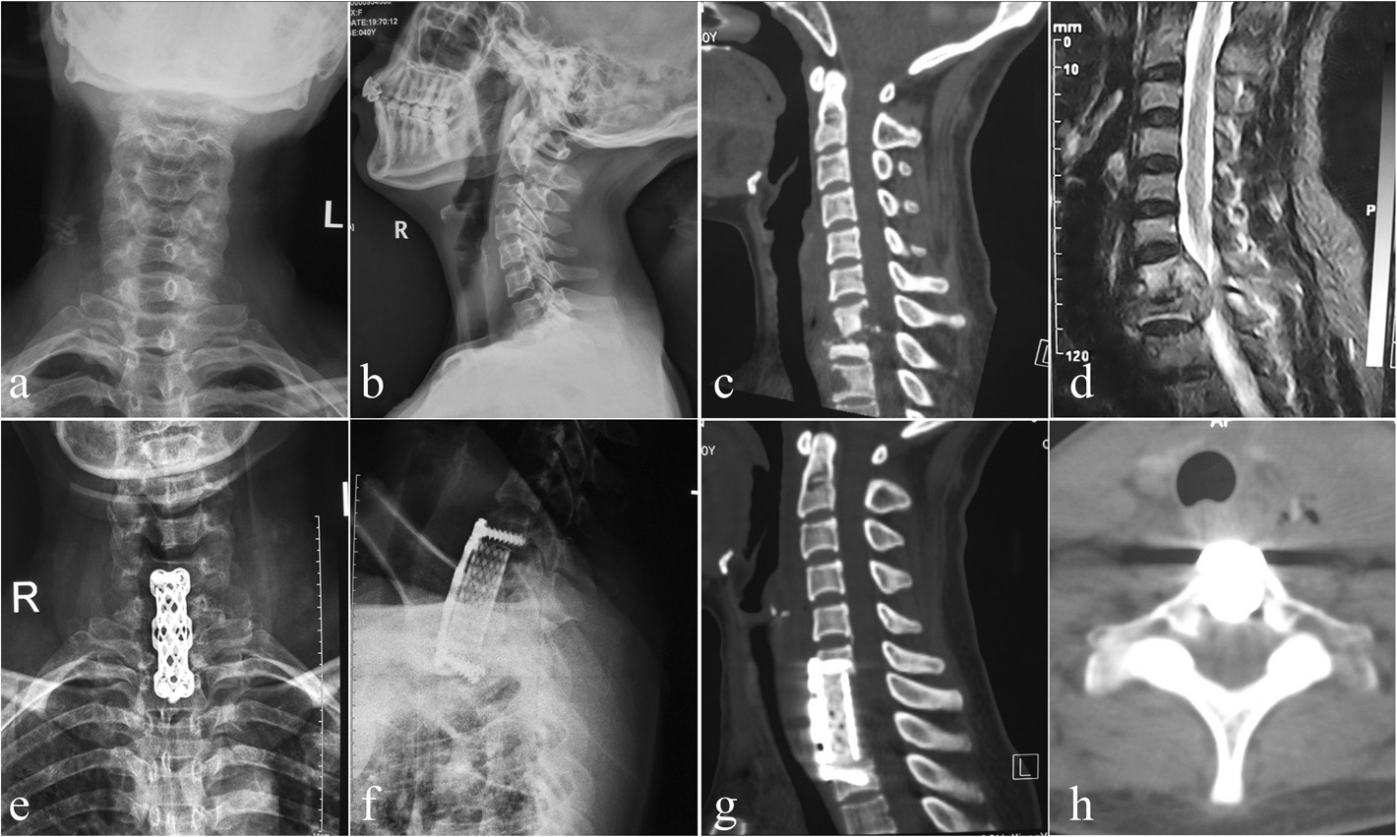
One patient in group A. Anterior-only approach was performed in a 40-year-old female with C7-T1 lesion and partial T2 destruction. **a**, **b** X-ray demonstrated sagittal instability and kyphosis. A pre-operative sagittal CT (**c**) and MRI (**d**) showed significant C7-T1 and partial T2 vertebral bodies’ destruction with mild kyphosis associated with epidural and paravertebral abscess formation, and the cervical spinal cord was severely compressed. A postoperative X-ray (**e**, **f**) indicated that the kyphosis got obviously improved; sagittal and coronal CT scan (**g**, **h**) showed satisfied focal clearance and decompression without graft and instrumentation-related complications and relapse of Pott’s disease at 12 months of post-operation

**Fig. 2:**
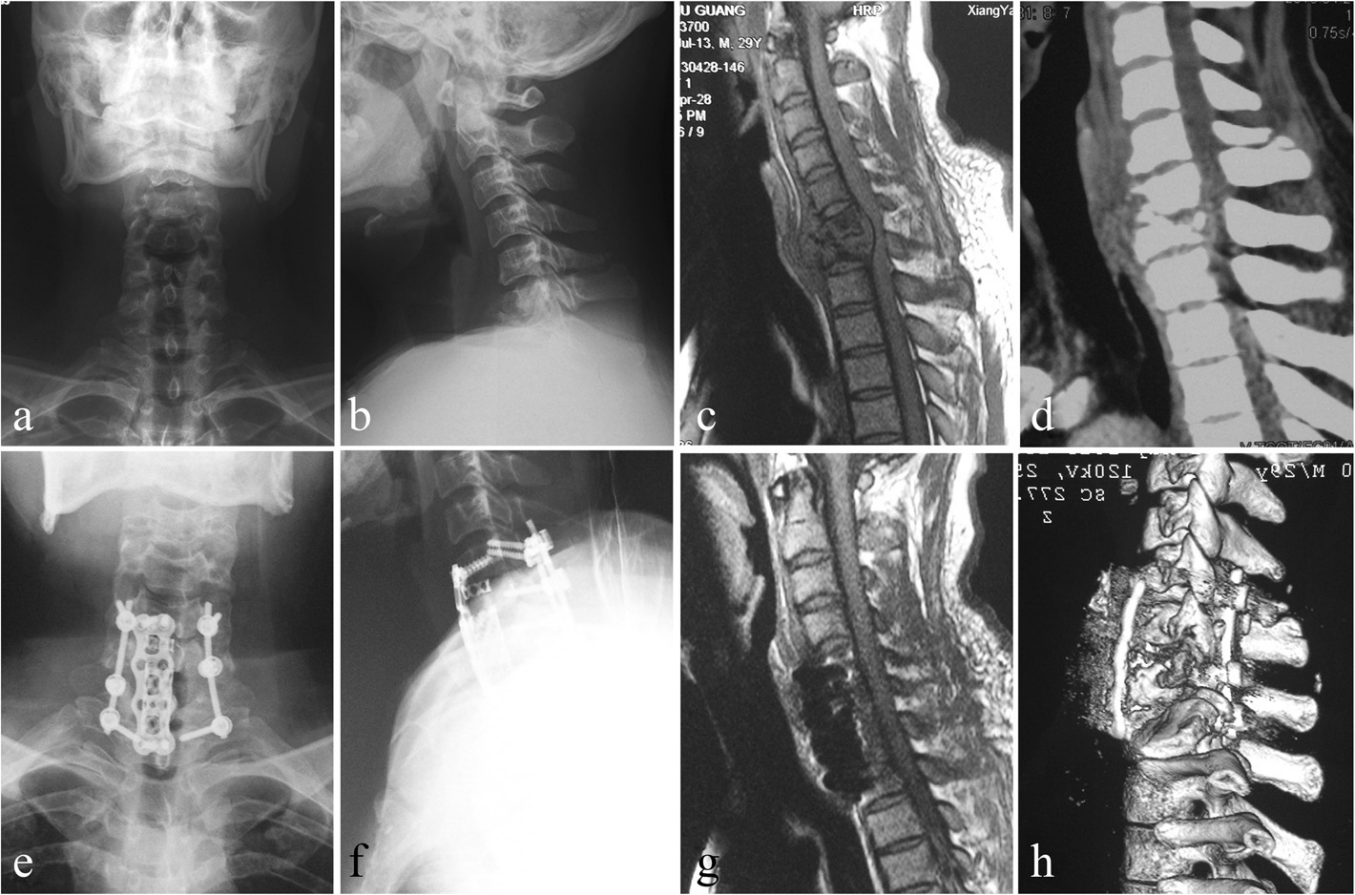
One patient in group B. Combined anterior and posterior approach was performed in a 30-year-old male. The pre-operative images (**a**–**d**) showed significant C6-7 vertebral bodies’ destructions with moderate kyphosis associated with epidural and paravertebral abscess formation, and the spinal cord was severely compressed. A postoperative X-ray (**e**, **f**) indicated that the kyphosis got obviously improved; CT scan and MRI (**g**, **h**) showed satisfied focal clearance and decompression and good fusion without relapse of Pott’s disease at the final follow-up

**Fig. 3:**
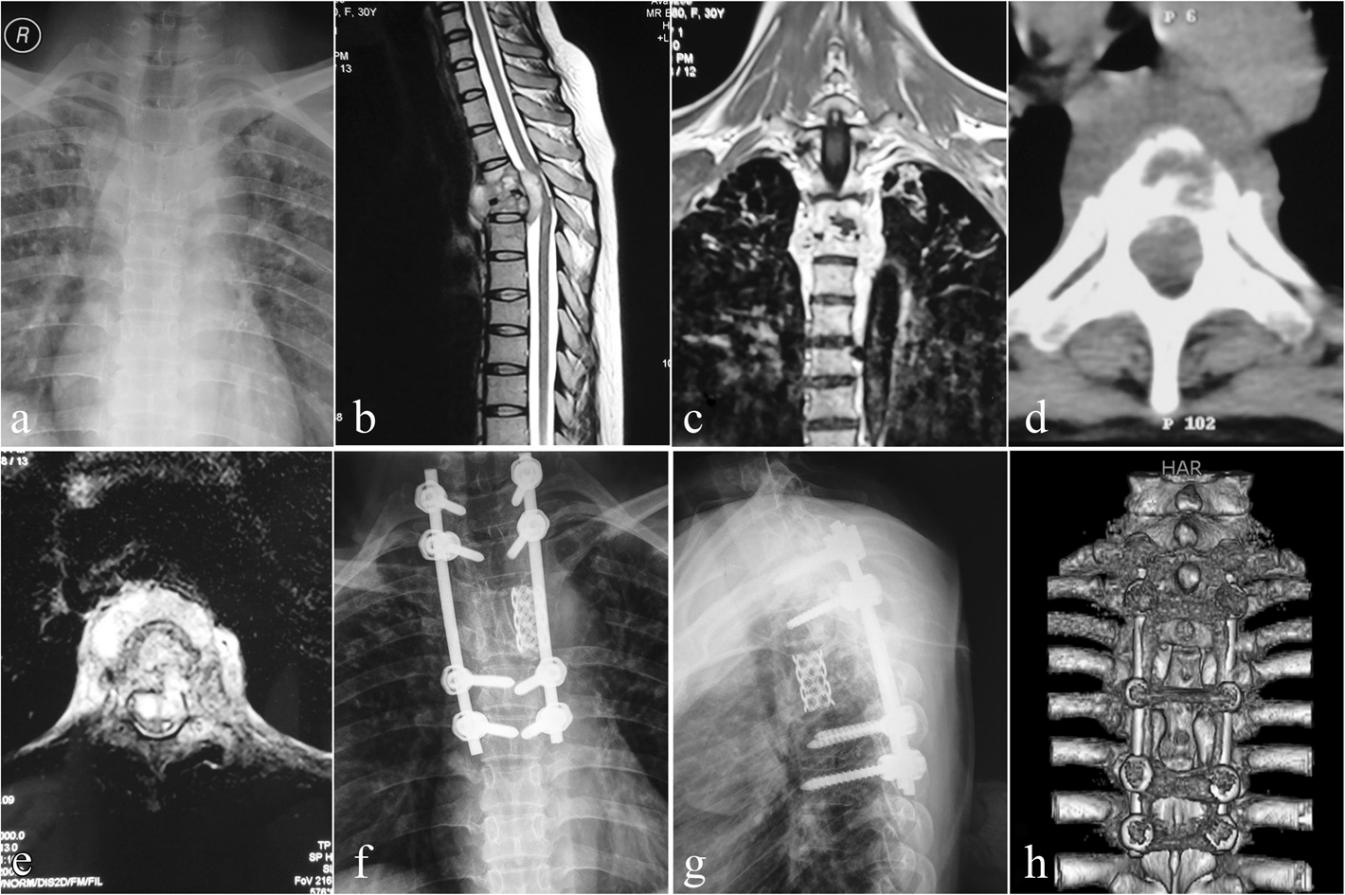
One patient in group C. Posterior-only approach was performed in a 30-year-old female. **a**–**e** The pre-operative imaging data showed significant T3–4 vertebral bodies’ destructions with mild kyphosis complicated with epidural and paravertebral abscess formation, and the spinal cord was severely compressed. The postoperative X-ray (**f**, **g**) indicated that the kyphosis got obviously improved; three-dimensional CT-scan (**h**) showed satisfied TMC and allograft fusion at 9 months of post-operation

From January 2000 to January 2013, 59 patients (34 males and 25 females, with an average age of 52.3 ± 5.1 years) with a diagnosis of CTSTB underwent surgery at our spinal center. Twenty patients (33.9 %) in group A underwent debridement, titanium mesh cage (TMC) bone fusion filled with autologous or allogeneic bone particles, and fixation by anterior-only approach operation; eighteen cases (30.5 %) in group B underwent combined anterior approach (debridement, bone fusion, and instrumentation) and posterior surgery (fixation plus posterolateral fusion); and twenty-one patients (35.6 %) in group C underwent the posterior transpedicular debridement, interbody and posterolateral bone fusion, posterior fixation without anterior debridement, and decompression by posterior-only approach. Clinical details of the surgical groups are presented in Table 1. The diagnosis of the CTSTB was guided by non-specific laboratory findings such as anemia, hypoproteinemia, elevation of erythrocyte sedimentation rate (ESR), and C-reaction protein (CRP) and by radiological findings including spinal X-ray films, computed tomography (CT), and magnetic resonance imaging (MRI) (Figs. 1, 2, and 3). Forty-five patients (76.3 %) were presented with neck pain, nuchal rigidity/restricted neck activity/torticollis, and anorexia and weight loss. And constitutional symptoms including night sweats and mild fever could be seen in all cases; cervical radiculopathy in 12 cases (5 in group A, 4 in group B, and 3 in group C), 20.3 %; spastic quadriparesis in 40 cases (13 in group A, 12 in group B, and 15 in group C), 67.8 %; and pulmonary tuberculosis and/or tubercular pleuritis/hydrothorax in 10 cases (3 in group A, 4 in group B, and 3 in group C),16.9 %. The kyphosis angle, ESR, and CRP of patients upon admission were an average of 21.4 ± 8.1, 48.2 ± 11.2 mm/h, 21.3 ± 9.8 mg/L in group A; 34.2 ± 17.5, 50.6 ± 10.3 mm/h, 19.8 ± 13.4 mg/L in group B; 33.4 ± 15.1, 48.4 ± 15.8 mm/h, 22.1 ± 10.5 mg/L in group C, respectively (Table 2). The classification of the American Spinal Injury Association (ASIA) was used to assess the neurological compromise function, and clinically neurological details of every group are described in Table 3. Written informed consent was obtained from all patients, and the study protocol was approved by the Xiangya hospital ethics committee. In practice, it is challenging to randomly select a surgical treatment method clinically. Therefore, in the present study, partial cases (infection invading T3–4) in group A were collected earlier while all cases in groups B and C were collected in more recent years (the emergence of pedicle screw technology and prevalence of posterior-only approach to spinal tuberculosis is promoting the development of combined anterior and posterior approach and even the fashion of simple posterior surgery in treatment of spinal tuberculosis). The same surgeons reviewed the surgical indications and performed the procedures.

**Table 1:** General data of this study

	Group A (*n*)	Group B (*n*)	Group C (*n*)	*P* value (*P*_A–B_/*P*_A–C_/*P*_B–C_)
Number of patients	20	18	21	
Male to female ratio	11:9	10:8	13:9	
Age in years (range)	53.4 ± 5.2 (19–70)	48.5 ± 4.3 (30–65)	55.1 ± 6.3 (20–73)	
Operation time (min)	170.5 ± 50.4	231.0 ± 30.2	190.4 ± 24.2	<0.05/>0.05/<0.05
Amount of bleeding (ml)	350.3 ± 99.3	448.3 ± 50.5	380.2 ± 44.5	<0.05/>0.05/<0.05
Hospitalization day (days)	8.2 ± 5.8	18.4 ± 8.7	12.4 ± 4.0	<0.05/<0.05/<0.05
Follow-up in months (range)	45.5 ± 6.1 (24–50)	42.6 ± 5.2 (26–60)	40 ± 3.8 (24–58)	>0.05/>0.05/>0.05
Mean fusion level (range)	3.8 (3–5)	4.8 (3–7)	4.0 (3–7)	<0.05/>0.05/<0.05

*PA*_–_ *B*, *PA*_–_ *C*, and *PB*_–_ *C P* value of comparison between groups A and B, A and C, B and C, by chi-square test, respectively

**Table 2:** Details in the kyphosis, ESR, and CRP of three surgical groups

Groups	Pre-operative kyphosis angle	Post-operation			Final follow-up			ESR (mm/h)		CRP (mg/L)	
		Kyphosis angle^※^	Angle correction	Correction rate (%)	Kyphosis angle ^*^	Angle lost	Lost rate (%)	Pre-op	3-month post-op^#^	Pre-op	3-month post-op^¥^
Group A	21.4 ± 8.1	7.3 ± 4.2	14.1 ± 5.3	65.9 ± 7.8	9.4 ± 4.8	2.1 ± 2.1	14.9	48.2 ± 11.2	10.4 ± 2.7	21.3 ± 9.8	7.8 ± 2.1
Group B	34.2 ± 17.5	5.0 ± 8.8	29.2 ± 5.3	85.4 ± 4.1	5.7 ± 7.0	0.7 ± 0.4	2.4	50.6 ± 10.3	9.7 ± 5.1	19.8 ± 13.4	6.1 ± 3.1
Group C	33.4 ± 15.1	6.8 ± 5.4	26.6 ± 10.8	79.6 ± 8.2	7.9 ± 5.0	1.1 ± 0.2	4.1	48.4 ± 15.8	10.2 ± 3.9	22.1 ± 10.5	6.2 ± 1.9
*P*_A–B_	<0.05	<0.05	<0.05	<0.05	<0.05	<0.05	<0.05	>0.05	>0.05	>0.05	>0.05
*P*_A–C_	<0.05	<0.05	<0.05	<0.05	<0.05	<0.05	<0.05	>0.05	>0.05	>0.05	>0.05
*P*_B–C_	>0.05	<0.05	>0.05	>0.05	>0.05	>0.05	>0.05	>0.05	>0.05	>0.05	>0.05

*ESR* erythrocyte sedimentation rate, *CRP* C-reaction protein, *pre-op* pre-operation, *post-op* post-operation, *PA*_–_ *B*, *PA*_–_ *C*, and *PB*_–_ *C P* value of comparison between group A and B, A and C, B and C, respectively

^※^Student–Newman–Keuls test, compared post-operation kyphosis angle with pre-operative in the three groups, *P*a = 0.0003, *P*b = 0.0001, *P*c = 0.0001; *Student–Newman–Keuls test, compared the final follow-up kyphosis angle with post-operation in the three groups, *P*a = 0.0523, *P*b = 0.2410, *P*c = 0.1321; ^#,¥^Student–Newman–Keuls test, compared ESR, CRP in 3 month post-operation with pre-operative in the three groups, *P* < 0.05

**Table 3:** Neurologic recovery according to ASIA in three groups

Pre-operation	Groups A/B/C	Final follow-up in group A^*^					Final follow-up in group B^#^					Final follow-up in group C^※^				
		A	B	C	D	E	A	B	C	D	E	A	B	C	D	E
A	0/2/1								2					1		
B	3/3/2			2	1					3					2	
C	3/6/6				3						6				3	3
D	10/6/9					10					6					9
E	4/1/3					4					1					3

*Wilcoxon signed rank test, compare with pre-operation, in group A, *P*a = 0.0021; comparison between group A and B, *P*_A–B_ > 0.05; ^#^Wilcoxon signed rank test, compare with pre-operation, in group B, *P*b = 0.0018; comparison between group B and C, *P*_B–C_ > 0.05; ^※^Wilcoxon signed rank test, compare with pre-operation, in group C, *P*c = 0.0020; comparison between group C and A, *P*_A–C_ > 0.05

## Methods

### Pre-operative procedure

The patients participating in this study had a clinical diagnosis of CTSTB and were administrated anti-TB drugs with the HRE (isoniazid, rifampin, and ethambutol) chemotherapy regimen, consisting of isoniazid (300 mg/day), rifampicin (450 mg/day), and ethambutol (750 mg/day) 2–4 weeks before surgery. Prudently, 11 patients (8 in group B and 3 in group C) with relatively severe kyphosis deformity were performed by halo traction with a weight of 1–4 kg pre-operatively. When the ESR, CRP, and the temperature returned to normal or had significantly decreased, and anemia and hypoproteinemia were rectified completely, it was time to carry out the procedure.

### Operative procedures (three approaches were chosen)

#### Group A: anterior-only approach

We performed a standard anterolateral cervical exposure extended caudally in a supine position, debridement, and TMC filled with autologous or allogeneic bone particles and internal fixation, which is similar to routine cervical anterolateral surgery, in a ‘long-neck’ patient with tuberculosis focus located in C7-T1. But we performed transsternal approach in patients with ‘short neck’ and/or the foci located in T1–4. The sternal portions of the sternocleidomastoid and infrahyoid muscles were located and cut roughly 2 cm from their sternal insertion. If access was limited by the manubrium sternum, the anterior aspect of the manubrium was exposed to the medial limits of the sternoclavicular joints. Careful finger dissection was used to free the posterior aspect of the manubrium, and then the bone was excised. This bone resection was limited by the sternoclavicular joints laterally and by the junction between the manubrium and the sternal body caudally. The posterior cortex of the manubrium was exposed using a high-speed drill to reduce the risk of injuring retrosternal structures. The powerful interclavicular ligament was cut with large scissors. The retrosternal fat and large vessels were then retracted together caudally and anteriorly to attain the anterior aspect of the thoracic vertebrae. The dissection of the large vessels was unnecessary and even appeared dangerous to us. During the decompression stage of the procedure, it was possible to improve exposure by excising the anteroinferior corner of the suprajacent vertebra. In every case, the procedure was completed by osteosynthesis using an anterior plate from the level overlying the one underlying the decompression [3]. We attempted to minimize the number of fused segments. If the height loss of the affected vertebrae was less than one-third of the vertebral body height, screws were inserted into the damaged vertebrae. If the destruction of the vertebrae was greater than one-third of the whole vertebral body, screws were inserted in the adjacent normal vertebrae. Bone grafting was carried out with suitable long bone grafts or titanium mesh cage mixed with 0.5 g isoniazid and 0.5 g streptomycin. Closed drainage and incision sutures were performed postoperatively (Fig. 1).

#### Group B: combined anterior and posterior approach

In the posterior approach, the patients were placed in a prone position with the halo traction of a weight of 2–3 kg during the operation. After routine exposure, a posterior fixation was performed in all patients. The technique for placement of the lateral mass screws (C5-C6) and pedicle screws fixation (C7-T6) was described by Magerl [6]. The instrumented fusion should span over three or four levels, at least reaching the upper and lower fusion vertebrae. When there was potential instability, the fixation segments should be extended. By posterior fixation, the kyphosis was tried to be corrected, but not excessively, because of front oppression of the spinal cord has not been lifted, and then the fixation was locked. In addition to instrumented fixation, a posterolateral fusion was performed in all cases by using a high-speed burr to decorticate the bilateral facet joints. Allograft particles were then packed into the decorticated facet joints. Finally, removal of halo traction, closed drainage, and incision sutures were performed postoperatively. If the general physical condition (cardiopulmonary function, nutritional status, etc.) of the patient may be tolerable, one-stage anterior operation should be advocated. Otherwise, implementation of the second-stage surgery was performed after 1–2 weeks. The above-mentioned anterior approach was performed in patients in the supine position (Fig. 2).

#### Group C: posterior-only approach

Posterior exposure and placement of internal fixation refer to the above-mentioned methods. Generally, we preferred longer segmental fixation, at least two levels superior and inferior to the level of decompression. The affected vertebra was often incorporated into the instrumentation system, if the upper part of the vertebral body was not destroyed by infection. Then a temporary rod on the mild side of the focus was stabilized to avoid spinal cord injury induced by instability of the spine during decompression and focal debridement. Costotransversectomy of the severe lesion segment was performed to drain prevertebral abscess and expose diseased vertebral bodies. According to the extent of spinal canal stenosis, partial laminectomy or total laminectomy was carried out at the decompression level to permit circumferential decompression. Thoracic nerve roots on the focal side were sacrificed for better exposure. If necessary, a facetectomy or pediculectomy was also performed. Then the abscess was drained by suction and curettages. Granulation tissue and bony sequestrum were debrided, and the cord was decompressed. Following completion of debridement and decompression, correction of the deformity was accomplished by installing contoured permanent rods with compression maneuvers. When manipulating compression procedure, it should be noticed that the spinal cord was not stretched or distracted. If space created after focal debridement was too large, autogenous or allograft bone would be implanted. Then autogenous bone or allograft was selected for posterior fusion at the segments that underwent decompression and focal debridement. Treatment with 1.0 g streptomycin and 0.2 g isoniazid was locally administered; drainage and incision sutures were performed postoperatively. The material debrided was sent for culture and histopathologic examination [2] (Fig. 3).

### Postoperative procedure

The drainage tube was pulled out when the volume of drainage was less 30 ml/24 h. The patients continued with oral aforementioned three-drug HRE chemotherapy for 2 months postoperatively. Afterwards, ethambutol was discontinued and patients then received 9- to 12-month regimens of HR chemotherapy (2 HRE/9–12 HR). All of the patients were examined clinically and radiologically at 1 week and 3, 6, and 12 months after surgery and then once a year. After 2 days of post-surgery, patients were allowed to ambulate with a hard cervical collar for at least 3 months in average postoperatively. Routine postoperative medical treatments were carried out, including dehydration treatment, conventional nerve nutrition, and prophylactic antibiotic administration. In addition, functional rehabilitation exercise was advised to all patients during the early stage of recovery.

### Follow-up index and statistical analysis

The average follow-up duration was 45.5 ± 6.1 months (ranges 24–50) in group A; 42.6 ± 5.2 months (ranges 26–60) in group B; 40.0 ± 3.8 months (ranges 24–58) in group C, respectively. The following indexes were recorded pre-operatively, postoperatively, and during the follow-up: (1) kyphosis angle; (2) neurological function by using the classification of the ASIA; (3) ESR and CRP; and (4) intraoperative and postoperative complications. Using SPSS 19.0 software (SPSS, Inc., Chicago, IL, USA), kyphosis angle, ESR, and CRP were statistically analyzed by Student–Newman–Keuls test pre-operatively, postoperatively, and/or during the follow-up, and neurological function was statistically analyzed by Wilcoxon signed rank test pre-operatively, and during the follow-up. Comparison of complications between the groups was statistically analyzed by chi-square test. Discrepancy of the normal distribution was analyzed by a rank-sum test with a significance level of 0.05 (Tables 2, 3, and 4).

**Table 4:** Complications of three groups in post-operation and follow-up

All complications	Group A (*n*)	Group B (*n*)	Group C (*n*)	*P* value (*P*_A–B_/*P*_A–C_/*P*_B–C_)
Dysphagia	3	4		0.687/0.107/0.037
Infection		2	1	0.218/1.000/0.586
Hardware-related complications	4	1		0.344/0.048/0.462
Pseudarthrosis	3			0.232/0.101/>0.05
Esophagostoma		1		0.474/>0.05/0.462
Cage subsidence	3			0.232/0.107/>0.05
Harvested axial pain			2	>0.05/0.488/0.490
Recurrence of tuberculosis			1	>0.05/1.000/1.000
Revision surgery	5	2		0.410/0.021/0.206
Total	18	10	4	0.027/0.000/0.024

*PA*_–_ *B*, *PA*_–_ *C*, and *PB*_–_ *C P* value of comparison between groups A and B, A and C, B and C, by chi-square test, respectively

## Results

### Clinical findings

The mean blood loss, operation time, and hospitalization time was 350.3 ± 99.3 ml, 170.5 ± 50.4 min, and 8.2 ± 5.8 days in group A; 448.3 ± 50.5 ml, 231.0 ± 30.2 min, and 18.4 ± 8.7 days in group B; and 380.2 ± 44.5 ml, 190.4 ± 24.2 min, and 12.4 ± 4.0 days in group C, respectively (Table 1). The histopathologic examination of the operative specimen revealed granulomatous infections consistent with tuberculosis.

### Laboratory data

The ESR and CRP of patients upon admission were shown with an average of 48.2 ± 11.2 mm/h and 21.3 ± 9.8 mg/L in group A; 50.6 ± 10.3 mm/h and 19.8 ± 13.4 mg/L in group B; and 48.4 ± 15.8 mm/h and 22.1 ± 10.5 mg/L in group C, respectively. And they got normal within 3 months in all patients. There was a statistical difference between pre-operative and 3 months follow-up (*P* < 0.05) (Table 2).

### Surgery-related complications

Wounds were unhealed with chronic infection or sinus formation in three cases (two in group B and one in group C). Seven patients (three in group A and four in group B) showed symptoms of mild dysphagia postoperatively, the symptoms disappeared after the patient was advised to have a liquid diet. Nine patients suffered from non-active pulmonary tuberculosis and mild tuberculous pleuritis pre-operatively, and eight patients had got healed without any particular therapy except routine chemistry treatment of CTSTB, while the remnant one got failed due to his poor compliance and was turned to a medical physician. Additional thoracocentesis plus closed drainage was performed in another patient complicated with moderate tuberculosis pleural effusion pre-operatively, and the patient harvested a satisfactory outcome. There were five cases (four in group A and one in group B) who suffered from hardware-related complications, such as displacement or settlement and fracture of fixation, and three patients in group A have complications with pseudarthrosis and cage subsidence. In group B, the esophagostoma of one patient was harvested and then the patient was administrated with fasting and water deprivation and neoplasty and got a good outcome. In group C, there were two patients presented with harvested axial pain and one of which suffered from recurrence of tuberculosis, which was later confirmed as multiple-resistant to anti-TB drugs. Susceptibility testing and personalized treatment benefit the patient at last. Revision surgery was performed in five cases in group A and two patients in group B due to hardware-related complications, pseudarthrosis, and cage subsidence potentially or actually leading to spinal instability and nerve compression (Table 4).

### Spinal deformity

The kyphosis angle of patients upon admission was showed with an average of 21.4 ± 8.1 in group A, 34.2 ± 17.5 in group B, and 33.4 ± 15.1 in group C. They significantly decreased to 7.3 ± 4.2° in group A, 5.0 ± 8.8° in group B, and 6.8 ± 5.4° in group C postoperatively (*P* < 0.05). The mean kyphosis angle was 9.4 ± 4.8° in group A, 5.7 ± 7.0° in group B, and 7.9 ± 5.0 in group C at final follow-up, whose correction of kyphosis and loss of correction were 14.1 ± 5.3 and 2.1 ± 2.1 in group A, 29.2 ± 5.3 and 0.7 ± 0.4 in group B, 26.6 ± 10.8 and 1.1 ± 0.2 in group C, respectively. They are still significantly improved in comparison to the pre-operative measurements (*P* < 0.05).

### Neural changes and pain

All patients had a different degree of improvement with aspect to neurological function at the final follow-up examination. Results were evaluated by ASIA classification and the specific details are in Table 3. *P* value of comparison of pre-operation with final follow-up by Wilcoxon signed rank test is 0.0021 in group A, 0.0018 in group B, and 0.0020 in group C. And all *P* values of comparison between the groups are more than 0.05, with no statistical significance. Twelve patients with cervical radiculopathy had immediate postoperative remission, and all patients had a relief of neck and back pain (Table 3).

### Bone fusion

The TMC filled with autologous or allograft bone particles and/or posterolateral fusion was performed in all patients. The presence of continuous, bridging, and bone trabeculae at the graft–host vertebral endplate junction was identified by CT scans (Figs. 1, 2, and 3). Pseudoarthrosis was determined by the absence of osseous trabecular bridging between the graft and host vertebra endplates and the presence of a lucent line at the graft–vertebral junctions in the sagittal reconstruction CT scans. In our study, all patients but three cases achieved bone fusion within 4–8 months after surgery, which were confirmed by two different surgeons based on the above criteria of radiological fusion. The three patients in group A were confirmed as pseudoarthrosis, and only one of which was performed by revision surgery due to potential neurologic impairment. Interestingly, the other two patients with pseudoarthrosis showed no discomfort and abnormality in clinical and radiography during the relatively long follow-up and avoided reoperation.

## Discussion

Spinal tuberculosis at the cervicothoracic junction rarely occurs. Cervicothoracic boundary is in a curvature from a mobile cervical lordosis to a rigid thoracic kyphosis, which significantly alters the biomechanics and the stability of the cervicothoracic junction. The loss or even collapse of the anterior column support at the cervicodorsal junction due to infection further aggravates the deformity. Instability results from destruction of the anterior column, and progressive kyphosis occurs due to toppling from the mobile cervical column over the fixed thoracic spine, in spite of the posterior spinal ligaments, musculature, and facet joints preventing deformity progression. Necrotic tissue and/or epidural abscess compressing the spinal cord, if not treated, can be complicated by paraplegia or even panplegia. However, small lesion and unobvious systemic and local symptoms make the early diagnosis difficult, attributed to various tissues and organs barrier, leading X-ray hard to find it during the beginning stage of CTSTB [2, 7, 12, 13].

### AO approach

It is generally recognized that the anterior approach directly addresses the clinically relevant pathology, radical debridement, more extensive decompression, and reconstruction of the anterior column, theoretically leading to improved rates of arthrodesis and a perfect clinical outcome. However, anterior exposure of the cervicothoracic junction, which is blocked by the thoracic bones, clavicle, costal bone, and superior mediastinum organs and macrovascular, indeed presents a significant challenge to the spine surgeon.

The transsternal approach popularized by Cauchoix and Binet [14] in 1957 and Hodgson et al. [15] in 1960 was used in adults for cervicodorsal disorders. But Hodgson subsequently abandoned the procedure because of severe complications following anterior decompression at this level. An et al. [7] reported a postoperative mortality of 33 % as a result of bleeding and sepsis following a modified transsternal approach. Mihir et al. [13] introduced a modified anterior cervical approach in anterior reconstruction and instrumentation of the cervicothoracic tubercular vertebrae and got good clinical outcomes; however, 7.14 % of all patients suffered from complications, such as recurrent laryngeal nerve palsy and pleural puncture, and 26 % were noticed with hypertrophic scar. Clinically and biomechanically, unfortunate complications are frequently encountered with anterior long-segment plate including screw and/or plate migration or displacement as well as breakage caused by the large cantilever forces generated at the caudal screw-plate junction [8, 16]. Operation time, hospitalization day, and amount of bleeding were shorter and less in group A than in groups B and C in our study; however, correction of kyphosis deformity and maintenance of correction in group A were inferior to groups B and C. Moreover, there were some complications, such as dysphagia in three cases, hardware-related complications in four cases, pseudarthrosis in three cases, and cage subsidence in three cases, which were probably attributed to anterior-related routine, and the surgery itself could not keep the long-term stability of spine. Therefore, we recommended that the indications of anterior-only (AO) approach includes (a) single-segment lower cervical lesion such as C6, C7, and the upper thoracic vertebrae, T1, especially in the long-neck patients; (b) infection and destruction confined to anterior column and abscess and necrotic tissue compressing the front of spinal cord; and (c) mild kyphosis deformity, less than 30° (Fig. 1).

### AP approach

The combined anterior and posterior approach is a well-established surgical technique, having been widely used for most cervicothoracic disorders with satisfactory clinical outcomes. Nottmeier et al. [17] and Wang et al. [12] described 360° reconstruction in cervical and cervicothoracic junction and suggested that unilateral surgical approach for treatment of cervical kyphotic deformity do have a theoretic decreased perioperative risk, but may sacrifice both degree and maintenance of deformity correction, and emphasize the degree of deformity correction and maintenance of deformity correction using 360° reconstruction. If anterior approach is combined with posterior approaches, it will significantly reduce the stress of plate and graft suffered and increase the stability of the cervicothoracic spine, reducing graft and/or fixation-related complications. McAfee et al. [18] reported 12 cases performed by TMC fusion and circumferential reconstruction that had got stable bone fusion and satisfactory kyphosis correction without dislodgement and loss of correction. However, these approaches do not only avoid the anterior approach-related complications but also increased another surgical scar, surgical trauma, and operation time and even bring unnecessary surgical complications, such as neck pain due to wide detachment and stretching of the paraspinal muscles resulting in ischemia, necrosis, and muscle denervation. In our study, patients in group B indeed harvested perfect clinical and imageology outcomes during the follow-up (Fig. 2); however, the longer operation and hospitalization time, more loss of bleeding, and anterior–posterior routine-related complications made the combined anterior and posterior (AP) approach not applicable to all CTSTBs, except multi-level segments lesion, severe destruction, and complex kyphosis deformity [19].

### PO approach

The development and maturation of pedicle screw technology have been promoting the popularities of spinal posterior surgery. However, the anterior column is prone to infection with *Mycobacterium tuberculosis*. Therefore, controversy about the posterior-only surgery in the treatment of CTSTB focuses on whether surgeons can perform complete focal debridement and anterior decompression in a so-limited visual field, whether posterior-only surgery affects anterior bony fusion, and whether it affects spinal stability due to posterior column destruction [12, 13].

Mehta and Bhojraj [20] presented impressive outcomes following posterior transpedicular debridement, bone fusion, and posterior fixation without anterior debridement in the patients at a high-risk transthoracic surgery result from their limited medical facility. Rath et al. [21] and Feyza et al. [22] delivered perfectly neurological recovery following posterior transpedicular debridement and instrumentation in patients with neurological deficit because of tuberculous spondylitis, which is similar to the best results obtained via anterior decompression. Zhang et al. [2] extended this technique in children with cervicothoracic tubercular kyphosis and got a good clinical efficacy. Theoretically, based on the scope of vertebral destruction and the extent of vertebral osteoporosis, the fixed segment via the posterior-only (PO) approach can be extended to increase stability in axial compression, flexion, lateral bending, extension, and clockwise/anti-clockwise rotations. Posterior long-segment instrumentation which is a rigid stabilization system can provide better kyphosis correction and is beneficial to the stress dispersion, which can effectively prevent implants failure and also achieve relief of pain due to spinal instability [22]. This approach creates sufficient operating space through resection of both sides of the facet joint, the diapophysis and even thoracic nerve roots, allowing operation on all surfaces of the vertebral body under direct visualization of the outside of the dura mater. This enables thorough removal of the focal TB and complete spinal decompression without injuring the spinal cord. It has been suggested that removing the TB focus via the posterior approach could cause intra-spinal infection and central nervous system complications such as TB meningitis [23]. However, none of the patients in our study developed TB meningitis, a finding consistent with other reports [2, 22]. But when lesions are confined to the anterior vertebral column, when there is severe multi-level vertebral TB, or when paraspinal abscesses are located far from the cervicothoracic vertebra, the AP approach should be given priority [14, 15, 21]. In the present study, the patients in group C got as similar clinical results as cases in group B and harvested the least complications. All patients with posterior-only approach got satisfactory correction and maintenance of kyphosis and neural functional recovery and avoided fixation and fusion-related complications.

The authors consider single-stage posterior-only transpedicular debridement, interbody fusion, and instrumentation as a radical treatment. Therefore, we must confirm the indications of the PO approach: (a) significantly deformed dura and nerve roots, the presence of spinal stenosis, and moderate kyphotic deformity, less than 50°; (b) the presence of significant vertebral collapse caused by bone destruction or multi-centric TB spondylitis, but with only one or two target segments, with lesions accessible through a posterior approach; (c) spinal cord compression by paravertebral/epidural abscess; (d) patients who had undergone several anterior operations, in whom the anatomical structure was unclear; (e) severe or progressive neurological dysfunction and persistent lower neck pain unresponsive to conventional therapy; and (f) elderly patients with complicated co-morbidities intolerant of extreme surgical intervention.

## Conclusion

The anterior-only surgery should be limitedly used for severe CTSTB. The combined anterior and posterior approach has satisfactory clinical and radiographic outcomes, but is complicated with a larger loss of blood and more complications, thereby leading longer hospitalization days, which should be reservedly performed for mild CTSTB, except multi-level segments lesion or complex kyphosis deformity. Compared to traditional surgery, the posterior-only surgery cannot only significantly improve clinical results but also obviously relieve postoperative complications, which are in line with the principles of minimally invasive surgery and is worthy of further research and promotion. However, the indication of surgery is relatively limited, which should be considered comprehensively for each individual. Of course, further study with a large number of patients and longer follow-up will be necessary.

## Abbreviations

AO: anterior-only approach
AP: combined anterior and posterior surgeries
ASIA: American Spinal Injury Association
ATT: anti-tuberculosis therapy
CRP: C-reaction protein
CT: computed tomography
CTSTB: cervicothoracic spinal tuberculosis
ESR: erythrocyte sedimentation rate
MRI: magnetic resonance imaging
PO: posterior-only approach
STB: spinal tuberculosis
TMC: titanium mesh cage
